# But CheckCif Said So!

**DOI:** 10.1063/4.0000898

**Published:** 2025-10-27

**Authors:** Mohammad T Chaudhry, Justin A Newman

**Affiliations:** Merck & Co., Inc., Rahway, NJ, USA

## Abstract

In this presentation we will dive into the world of inclusion complexes and their often complicated, confusing, and heavily disordered structures. This presentation will look at some recent literature and point out pitfalls of refining these structures and will place particular emphasis on the traps that reviewers have fallen prey to. And finally, some best practices for crystallographers, reviewers, and editors will be presented.

